# Conventional and Emerging Meat Processing Techniques for Improved Shelf Life and Quality

**DOI:** 10.3390/foods15010125

**Published:** 2026-01-01

**Authors:** Lubomír Lapčík, Barbora Lapčíková, Robert Gál

**Affiliations:** Department of Foodstuff Technology, Faculty of Technology, Tomas Bata University, Zlin, Nam. T.G. Masaryka 5555, 76001 Zlín, Czech Republic; lapcikova@utb.cz (B.L.); gal@utb.cz (R.G.)

The global meat industry continues to face increasing demands for longer shelf life, improved quality, and enhanced food safety, driving innovation across both conventional and emerging processing technologies. This Special Issue brings together a collection of original research and reviews that illuminate advancements in thermal and non-thermal processes, natural preservation strategies, biotechnological tools, intelligent quality assessment, and safety-focused reformulations. Collectively, the contributions provide a comprehensive overview of how modern processing interventions can protect microbial stability, inhibit chemical deterioration, and preserve the sensory and nutritional integrity of meat and related products. The total number of submissions to this Special Issue was nine, of which three were rejected and one was withdrawn. The following are the five accepted and already published papers:

Zhang et al., 2025 provide an in-depth review of microbial control strategies in low-temperature meat processing, highlighting the growing relevance of non-thermal sterilization and natural antimicrobials. Their work underscores how technologies such as cold plasma, high-pressure processing, and targeted biocides can maintain product quality while reducing energy consumption and meeting consumer expectations for clean-label foods.

Liu et al., 2025 explore the potential of natural plant extracts as inhibitors of harmful processing contaminants, comparing cumin from three geographical origins for its ability to suppress β-carboline heterocyclic amine formation in smoked meat. Their findings demonstrate that cumin—particularly the Hetian variety with the highest flavonoid content and antioxidant activity—can effectively reduce HCA accumulation in both model systems and meat patties, offering a promising natural strategy for improving the safety of smoked meat products.

In the context of seafood preservation, Waris and Pilavtepe-Celik 2025 evaluate aloe vera gel as an eco-friendly antioxidant coating for cold-stored sea bass slices. Treatments with 75% and 100% gel successfully slowed lipid oxidation and extended shelf life without compromising sensory quality, illustrating the potential of plant-based coatings as sustainable alternatives to synthetic antioxidants.

Innovations in digital quality assessment are presented by Liu et al., 2025, who introduce BBSNet—a lightweight few-shot learning model designed to classify pork freshness with limited training data. By integrating batch channel normalization and fine-grained feature extraction, the model achieves high accuracy while substantially reducing data requirements, bridging the gap between laboratory indicators and real-time industrial applications.

Finally, Ducic et al., 2025 examine the safety and quality of nitrite-free Sremska dry fermented sausage subjected to mild pasteurization. Their study confirms that eliminating nitrite, when combined with targeted heat treatment and starter cultures, can maintain microbial safety, control biogenic amine accumulation, minimize lipid oxidation, and preserve sensory acceptability, offering a viable pathway for producing high-quality nitrite-free fermented meats.

As a conclusion, the contributions in this Special Issue illustrate the dynamic evolution of meat processing, from natural antioxidants and antimicrobial interventions to digital assessment tools and reformulated products that address both safety and consumer health concerns. We trust that this collection will serve as a valuable resource for researchers, industry professionals, and policymakers seeking to advance meat preservation technologies and promote sustainable, high-quality products for global consumers.

